# Tracheal extubation and reintubation of the critically ill - challenges coming and going

**DOI:** 10.1186/s13054-015-0841-9

**Published:** 2015-03-27

**Authors:** Matteo Parotto, Richard M Cooper

**Affiliations:** Department of Anesthesia, University of Toronto and University Health Network, (Toronto General site), 200 Elizabeth St, Toronto, ON M5G 2C4 Canada

## Abstract

Tracheal reintubation is a common event in critical care. Elmer and colleagues provide the first comparison of complication rates of initial and subsequent reintubation(s) during the same hospitalization. Their work shows an increased risk of complications associated with reintubation, in particular hypoxemia and hypotension, reminding us to be cautious with patients having minimal reserve and potentially altered airway anatomy.

See related research by Elmer *et al.*, http://ccforum.com/content/19/1/12

## Commentary

Previously in *Critical Care*, Elmer and colleagues [1] presented their investigation on peri-procedural complications and difficulties managing the airways of patients requiring emergent reintubation. This represents an important domain in the care of the critically ill - the need for reintubation is frequent, challenging and independently associated with increased morbidity and mortality [2]. Concerns of clinicians dealing with extubation and subsequent reintubation are related to our inability to accurately predict when extubation will succeed and the challenges we confront in attempting to safely re-establish the airway. Airway obstruction may result from altered anatomy following difficult or prolonged intubation [3]; inadequate ventilation may be a consequence of deconditioning after prolonged immobility, or an imbalance between the energy supply and demand and technical challenges or physiologic disturbances of varied causation and consequence.

Elmer and colleagues’ retrospective analysis of a prospective registry of in-hospital emergency airway management is the first to compare the technical difficulty or procedural complication rates of initial and subsequent intubation events, offering some novel information and raising important questions.

Their observational work confirms that reintubation outside of the operating room during the same hospital admission is common in critically ill patients, occurring in 14% of cases, consistent with other reports [4]. In their cohort, reintubation was associated with a higher rate of procedural complications, in particular hypotension and hypoxemia. Furthermore, there were more patients with aspiration, dental trauma and esophageal intubation during the reintubation. A number of factors may contribute to the need for reintubation as well as a precarious response to it. These include existent hypoxemia, muscle weakness, diaphragmatic dysfunction, ineffective cough, altered sputum production, impaired mucociliary clearance and delayed gastric emptying [5,6]. Reduced respiratory and hemodynamic reserve may diminish tolerance to apnea or result in an exaggerated hemodynamic response to induction medications, laryngoscopy and intubation as observed by Elmer and colleagues and others.

Interestingly, the complications observed at the time of reintubation did not appear related to challenges in airway management. Although no difference was observed in the Cormack Lehane grades or the number of intubation attempts between the initial event and the final event, the trend toward more frequent esophageal intubations and dental trauma may reflect unperceived difficulty.

Prior airway management difficulties may in themselves be a risk factor for reintubation. This could be reflected by the more common use of adjuncts to direct laryngoscopy during the initial intubation, compared with patients not requiring reintubation (11% versus 1%), or the more frequent requirement of three or more attempts during their initial event compared with patients not requiring reintubation (11% versus 6%). Complex airway management may predispose to extubation failure because of induced laryngeal edema and/or other anatomical abnormalities [3]. This was previously reported to occur in about 5 to 15% of patients, being more common in women, and correlated to reason for admission, duration of mechanical ventilation and traumatic/difficult intubation [7,8].

The study raises a number of unanswered questions. First, can tolerance of apnea be improved with better methods of 'pre-oxygenation', such as high-flow nasal oxygenation or non-invasive ventilation [9,10]? Second, does greater reliance on topical anesthesia and avoidance of sedation and muscle relaxation reduce procedural complications or prolong and frustrate intubation efforts [11]? Third, if sedation or unconsciousness is induced, can the choice of medication reduce the likelihood of hypotension [12]? Fourth, does the cuff-leak test [8] or airway ultrasound [13] reliably predict patients with periglottic edema? If so, should extubation be delayed or will this only worsen airway swelling? Should we routinely give pre-extubation steroids (or selectively in those with evidence of edema) [14]? Fifth, should alternative intubation techniques such as videolaryngoscopy be resorted to as a primary rather than rescue plan [15]? Sixth, if reintubation difficulty is defined by the number of attempts, will this result in fewer but longer efforts with attendant risks?

We should not forget that extubation of the critically ill patient is elective and will frequently require reintubation, both of which need to be carefully planned.
